# The Effect of Gelatin Source on the Synthesis of Gelatin-Methacryloyl and the Production of Hydrogel Microparticles

**DOI:** 10.3390/gels9120927

**Published:** 2023-11-24

**Authors:** David Grijalva Garces, Luise Josephine Appoldt, Jasmin Egner, Nico Leister, Jürgen Hubbuch

**Affiliations:** 1 Institute of Functional Interfaces, Karlsruhe Institute of Technology, 76344 Eggenstein-Leopoldshafen, Germany; 2 Institute of Process Engineering in Life Sciences Section IV: Biomolecular Separation Engineering, Karlsruhe Institute of Technology, 76131 Karlsruhe, Germany; 3 Institute of Process Engineering in Life Sciences Section I: Food Process Engineering, Karlsruhe Institute of Technology, 76131 Karlsruhe, Germany

**Keywords:** biomaterials, bloom value, gelatin, GelMA, hydrogel, microfluidics, microparticle

## Abstract

Gelatin methacryloyl (GelMA) is widely used for the formulation of hydrogels in diverse biotechnological applications. After the derivatization of raw gelatin, the degree of functionalization (DoF) is an attribute of particular interest as the functional residues are necessary for crosslinking. Despite progress in the optimization of the process found in the literature, a comparison of the effect of raw gelatin on the functionalization is challenging as various approaches are employed. In this work, the modification of gelatin was performed at room temperature (RT), and eight different gelatin products were employed. The DoF proved to be affected by the bloom strength and by the species of gelatin at an equal reactant ratio. Furthermore, batch-to-batch variability of the same gelatin source had an effect on the produced GelMA. Moreover, the elasticity of GelMA hydrogels depended on the DoF of the protein as well as on bloom strength and source of the raw material. Additionally, GelMA solutions were used for the microfluidic production of droplets and subsequent crosslinking to hydrogel. This process was developed as a single pipeline at RT using protein concentrations up to 20% (*w*/*v*). Droplet size was controlled by the ratio of the continuous to dispersed phase. The swelling behavior of hydrogel particles depended on the GelMA concentration.

## 1. Introduction

Hydrogels are polymeric networks with a high water-binding and retaining capacity. Since the backbone of the hydrogels is crosslinked polymers, the structural stability of the hydrogel is preserved in aqueous phase [1]. These properties enable the transport of dissolved molecules within the physical structure which can be beneficial for a variety of biotechnological applications such as the immobilization of enzymes [2] and microorganisms [3,4] in bioreactors, as well as cell culture for studies of cellular metabolism [5]. For these diverse purposes, advanced manufacturing strategies are applied for the creation of defined physical structures such as microparticles in microfluidics [6] and tissue models in bioprinting [7].

A suitable biomaterial for the production of hydrogels is gelatin, which is extracted from collagen [8]. Furthermore, the molecular weight and molecular weight distribution of gelatin not only depend on the sources but also on the processing conditions such as treatment time, pH, and temperature. Gelatin extracted in acidic media, and media extracted using alkaline milieus, shows isoelectric points (IEP) at pH 8–9, and pH 4–5, respectively [9,10]. After processing, the protein backbone retains sites for cell adhesion as well as for enzymatic cleavage such as those present in collagen [8]. A challenging property of gelatin for certain applications is the transition of the gelatin solution to a gel below a physiological temperature. A way to handle the limited structural stability of hydrogels at elevated temperatures is the formation of covalent bonds between the proteins. For this purpose, gelatin is functionalized to gelatin methacryloyl (GelMA). The methacrylate and methacrylamide residues present in GelMA enable the creation of crosslinked networks via photopolymerization [11]. The first draft of the process was proposed by Van den Bulcke et al. [11]. The study included the addition of methacrylic anhydride (MAA) to the gelatin solution in phosphate-buffered saline (PBS) at pH 7.5 under stirring at 50 °C. Significant progress has been made by research groups to identify the effect of process parameters on the resulting degree of functionalization (DoF) of GelMA. Lee et al. [12] and Shirahama et al. [13] have presented a thorough characterization of the reaction using porcine gelatin. In these studies, the MAA-to-gelatin ratio was significantly reduced by using carbonate bicarbonate (CB) buffer at pH values above the IEP of porcine gelatin. This enhancement is due to the fact that free amino groups are not charged. Additionally, Shirahama et al. [13] studied the derivatization of gelatin in a temperature range from 35 to 50 °C with no difference in the produced DoF. Our previous study complemented the findings of both groups by producing porcine GelMA at room temperature (RT) while keeping the MAA-to-gelatin ratio at the same value [14]. Despite the improvement of the synthesis process concerning porcine GelMA, more work is required to compare the effect of raw material on the final product. To the best of our knowledge, a wide range of raw materials including a variation in species and bloom strength have only been reported once [15]. However, the used synthesis buffer was composed of 0.1 M CB buffer, lower than the optimum reported by Shirahama et al. [13]. Further studies have compared the use of porcine and bovine gelatin pairwise. However, making a comparison across studies is challenging. This is because the applied methods vary in terms of buffer composition and pH (PBS at pH 7.4 [16,17,18] or CB at pH 9 [15,19,20]), as well as buffering capacity (0.1 M [15,19] or 0.25 M CB [20]).

As GelMA contains cell adhesion sites, hydrogel microparticles can be used for cellular expansion and differentiation. Commonly used methods for the expansion of adherent cell types are based on the use of tissue culture (TC) flasks. This limits the production of large quantities of cells as the required physical space increases linearly with the number of required flasks. In contrast, a significant advantage is shown by the expansion of cells using microcarriers. Hydrogel microparticles offer a high growth surface-to-volume ratio and can be implemented into stirred bioreactors [21]. The application of GelMA when compared to underivatized gelatin has the advantage that crosslinking can be performed via photopolymerization in a single stage when producing hydrogel microparticles. In contrast, particle production with gelatin requires multiple stages [22]. The challenging property of GelMA solutions, however, is the sol–gel transition below 30 °C. This issue has been addressed in the literature by using relatively low concentrations of the protein, i.e., below 10% (*w*/*v*) [23,24,25], or by heating the entire microfluidic systems [25,26]. In the first part of this work, we apply the previously presented method to produce GelMA at room temperature. To characterize the effect of the raw material on the produced GelMA, we use a wide range of gelatin products. Porcine gelatin of five different products was tested. The samples included two separate batches of the most commonly studied gelatin product, i.e., porcine gelatin, 300 g bloom strength. Additionally, fish gelatin as well as two bovine products with varying bloom values were incorporated into the study. Furthermore, the produced GelMA was used for the formulation of hydrogels. The elasticity as a function of the source of the raw material was characterized. As a second part of the study, fish and porcine GelMA were used for the microfluidic production of droplets and the subsequent crosslinking to hydrogel microparticles. The manufacturing of microparticles was performed on a single pipeline at room temperature. The resulting droplet size was controlled by variation in the feed ratio of continuous to disperse phase, as well as by variation in GelMA type and concentration. In addition, the swelling behavior of hydrogel microparticles in aqueous media was determined.

## 2. Results and Discussion

### 2.1. GelMA Synthesis and Characterization

**Figure 1:**
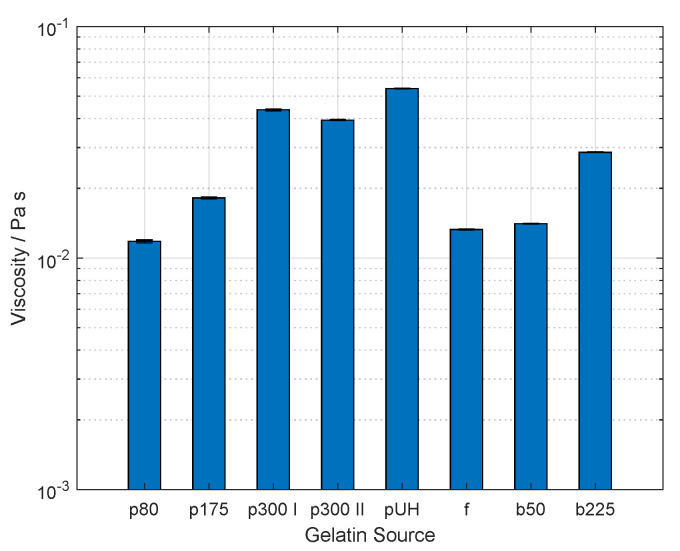
Viscosity of solution comprising gelatin at 10% (*w*/*v*) in reaction buffer, i.e., 0.25 M carbonate bicarbonate (CB) buffer, and 0.25 M urea, measured at room temperature. Sample nomenclature is provided in Table 1. The viscosity increased with increasing bloom strength of both porcine and bovine gelatin. Additionally, the viscosity of gelatin solutions prepared with the same product but different batches, i.e., p300 I, and p300 II, showed a significant difference. Statistically significant differences between the viscosity values were found between all data sets (p<0.05). Values are shown as mean and standard deviation. Each gelatin solution was tested three times from independently prepared samples.

As demonstrated previously, the dissolution of porcine gelatin of 300 g bloom strength in urea-containing buffer was possible solely under stirring at room temperature [14]. This method was applicable for dissolving porcine gelatin of various bloom strengths, and two different gelatin products from bovine tissue. All used gelatin products are listed in Table 1. The dissolution at room temperature was due to the fact that urea disrupts protein–protein hydrophobic interactions and causes gelatin to unfold to coils in solution [27,28,29]. Even though gelatin from cold-water fish does not form a physical gel above 5 to 10 °C due to the lower content of proline and hydroxyproline [9,30], the same synthesis buffer was used for the sake of comparability during the synthesis of GelMA. As the rheological behavior of protein solution affects the distribution of reactants during a stirred reaction, the viscosity of the gelatin solutions in the synthesis buffer was measured. Figure 1 provides the corresponding results.

The viscosity of solutions containing porcine gelatin increased significantly from 11.80 ± 0.17 mPa s to 53.93 ± 0.25 mPa s with increasing bloom strength of the gelatin product. The latter value was shown by the solution using a porcine source labeled as gelatin with ultrahigh (UH) gel strength by the supplier. Two batches from the same product with a bloom strength of 300 g were acquired and used for the measurements of viscosity. The solution produced with p300 I and p300 II gelatin showed a viscosity of 46.63 ± 0.42 mPa s and 39.43 ± 0.25 mPa s, respectively. These two values were significantly different (p<0.05). Fish gelatin solution showed a viscosity of 13.26 ± 0.06 mPa s. The viscosity of the solutions comprising bovine gelatin showed an increase in viscosity with increasing bloom strength of the product from 14.07 ± 0.06 mPa s to 28.60 ± 0.10 mPa s. Statistically significant differences between the viscosity values were found between all data sets (p<0.05). The increase in viscosity of gelatin solutions with increasing bloom strength is in accordance with other studies [30,31]. This is because the bloom value correlates with the molecular weight (MW) of gelatin [9]. Therefore, an increasing molecular weight leads to increasing intramolecular friction and to a higher amount of entanglements of proteins in solution, and, thus, higher viscosity [32,33]. In the case of gelatin pUH, no bloom value is stated by the producer. However, it was assumed that the MW is higher than that of gelatin p300 I and p300 II due to the ultrahigh gel strength. This was confirmed by the higher viscosity of the solution. Similarly, no bloom value is provided for fish gelatin. This is because the determination of gel strength is performed following a standardized method at 21 °C. Therefore, no bloom value can be measured for this product. The viscosity of the fish gelatin solution was around the values of viscosity of porcine gelatin p80 and bovine gelatin b50. Thus, the MW of fish gelatin was around the same magnitude as that of bovine and porcine gelatin of lower bloom strengths, as has been observed in the literature [30].

**Figure 2:**
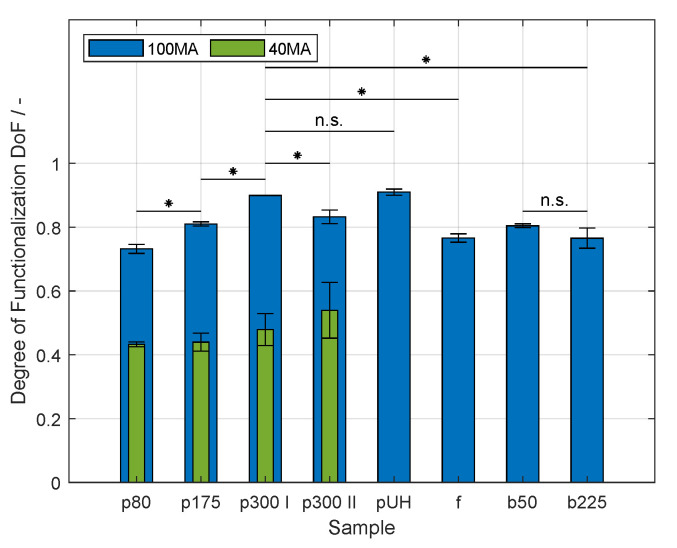
Degree of functionalization (DoF) of produced gelatin methacryloyl (GelMA). Sample nomenclature regarding the used raw materials is provided in Table 1. The DoF was determined by the trinitrobenzenesulfonic (TNBS) acid method [38]. At a methacrylic anhydride (MAA)-to-gelatin ratio of 100 μL g^−1^ (100 MA), asterisks denote a significant difference between synthesized samples (p<0.05). No significant differences are denoted with the abbreviation n.s. (p>0.05). Moreover, the DoF of porcine GelMA decreased significantly with decreasing MAA-to-gelatin ratio from 100 to 40 μL g^−1^ (40 MA) (p<0.05). At a MAA-to-gelatin ratio of 40 μL g^−1^ (40 MA), no significant differences regarding the DoF were proven (p>0.05). These differences are not shown for the purpose of clarity. Values are shown as mean and standard deviation. The functionalization of each gelatin type was carried out separately three times. The DoF of each batch was determined.

Although it has been mentioned in the literature that the DoF of GelMA might vary when different types of gelatin are used [34], not many reports have been presented on this topic. In this study, GelMA was synthesized using different gelatin products with varying species of origin and various values of bloom strength. Table 1 provides relevant information on the tested products. The DoFs of the produced samples are shown in Figure 2. In the first part of the study regarding the synthesis of GelMA, the previous method using a urea-containing buffer to process gelatin at room temperature was simplified [14]. In contrast to the said study where MAA was continuously fed during the reaction, the complete amount of reactant was added at the beginning of the reaction in the present study. Additionally, the reaction time was shortened to 60 min. The GelMA sample p300 I was produced using the same gelatin product and batch. At a MAA-to-gelatin ratio from 100 μL g^−1^ (100 MA), the DoF exhibited a value of 0.899 ± 0.010. This value is not significantly different from the data shown previously with a value of 0.963 ± 0.027 [14]. Reaching a similar DoF in spite of the reduction in reaction time is comparable to the study by Shirahama et al. [13], as it was shown that the reaction is completed within 60 min when the complete volume of MAA is added at the starting point. As mentioned above, porcine gelatin with a bloom strength of 300 g has been widely studied for the production of GelMA [18,20,35]. Moreover, batch-to-batch variability is known to be a drawback of naturally derived polymers [36,37]. To test the effect of such inconsistencies, a second batch of the same product was used to synthesize GelMA p300 II. The DoF showed a value 0.832 ± 0.021 at 100 MA, significantly lower than that of GelMA p300 I (p<0.05). Furthermore, the feasibility of using the developed method with porcine gelatin of varying bloom strength and the effects thereof were studied at 100 μL g^−1^ (100 MA). The DoF values of GelMA were 0.732 ± 0.014, and 0.810 ± 0.007 for the samples produced with gelatin of lower bloom strength, i.e., samples p80-100 MA, and p175-100 MA, respectively. These values differed significantly from each other and from the DoF of GelMA p300 I-100 MA (p<0.05). Additionally, porcine gelatin with ultrahigh gel strength was modified to GelMA with a DoF of 0.910 ± 0.010. However, these data did not differ significantly from the data of the sample p300 I-100 MA.

Gelatin p300 I and p300 II were derived from porcine skin with an acidic treatment (Type A) due to the high fat content of the tissue [10,30]. Individual differences within a species could lead to differences in MW and MW distribution. Additionally, slight differences in processing could also affect the properties of porcine gelatin as has been shown by Duconseille et al. [39]. The study showed that minor differences in the raw material and processing steps have a significant impact on the biochemical composition. In the reaction of gelatin to GelMA, the organic compound MAA is added to the aqueous gelatin solution. As both liquids are not miscible, thorough stirring is required to disperse the reactant to fine droplets. This issue has been addressed in the literature [13,35,40]. Hence, gelatin shows surface active properties leading to the adsorption of molecules to the created interface [41,42]. During stirring, MAA droplets are formed, which then collapse at different rates depending on the adsorption rate of gelatin to the interface and on the stabilization mechanism of the droplets. For instance, Shirahama et al. [13] mentioned that it was not feasible to evenly distribute MAA within a 1% (*w*/*v*) gelatin solution as not enough protein was in solution to stabilize the MAA droplets. Furthermore, the adsorbed amount is dependent on the MW and MW distribution [43]. Additionally, the MW of the adsorbed protein affects the stabilization mechanism of the created droplets [41,43]. As the reaction took place in a buffered solution at pH 9, around the isoelectric point (IEP) of porcine gelatin, the stabilization mechanism is mostly steric. The magnitude of stabilization as well as the amount of adsorbed protein both increase with increasing MW. Consequently, the stabilization provided by gelatin of higher MW, i.e., higher bloom value, yields a higher interface and therefore a higher reaction rate leading to higher values of DoF. A study was presented by Aljaber et al. [15], where porcine gelatin of 300 g as well as 175 g bloom strength was used to produce GelMA, and showed a higher DoF for the material of higher bloom value, which is in accordance with the presented data in this study.

The possibility to transfer the developed approach to raw materials other than porcine gelatin was tested using fish gelatin from cold water as well as two bovine gelatin products with different gel strengths. The buffer system and the processing at room temperature proved to be applicable to fish and bovine gelatin. As mentioned above, the presence of urea in the buffer inhibits the formation of helical structures of the gelatin from bovine tissue, i.e., the transition from solution to a gel. At a MAA-to-gelatin ratio from 100 μL g^−1^ (100 MA), the DoF of fish GelMA showed a value of 0.766 ± 0.013. Furthermore, the effect of various values of bloom strength was studied using bovine gelatin at 100 μL g^−1^. The DoF values of GelMA were 0.804 ± 0.006, and 0.765 ± 0.013 for the samples produced with gelatin of lower bloom strength, i.e., samples b50-100 MA, and b225-100 MA, respectively. The DoF of both bovine GelMA samples as well as fish GelMA differed significantly from the DoF of GelMA p300 I-100 MA (p<0.05). The effect of increasing bloom strength on the resulting DoF of bovine GelMA was not significant. As fish gelatin is extracted using acidic media [10], the IEP of the protein is similar to that of porcine gelatin. The determined DoF of GelMA f-100 MA is in the same range as the DoF of porcine GelMA with the lowest bloom strength, i.e., p80-100 MA. This comparable result is due to the fact that the MW of fish gelatin lies around the MW of gelatin p80, as was mentioned above regarding the results of the viscosity of both gelatin solutions. Hence, the stabilization of MAA droplets could take place at a similar magnitude. It has been shown by Lee et al. [12] that the reaction is most effective when the free amino groups are not charged; therefore, the pH during the reaction as well as the IEP of gelatin plays a significant role during the production of GelMA. In the literature, a higher DoF of bovine GelMA compared to that of porcine GelMA has been reported [16,18]. Both studies performed the reaction using phosphate-buffered saline, leading to the crucial difference in the surface charge of both proteins. The IEP of bovine gelatin lies around pH 4–5 due to the alkaline pre-treatment of bovine tissue where asparagine and glutamine are converted to aspartic acid and glutamic acid, respectively, [9,10]. Therefore, at pH 7 the reaction rate of bovine GelMA is much higher than the rate of porcine gelatin. Further studies producing GelMA using CB solutions have shown similar DoF values for porcine and bovine products. Lee et al. [19] prepared GelMA using porcine gelatin with 175 g bloom strength and bovine gelatin with 225 g. Both samples showed similar DoF values, which is in accordance with the presented study. Aljaber et al. [15] prepared GelMA using porcine gelatin with 300 g bloom strength, which had a higher DoF than the GelMA produced from bovine gelatin. Although the bloom strength of the bovine protein was not stated in that study, the results are in accordance with the results shown in this manuscript. As mentioned above, MAA and aqueous gelatin solutions are not miscible, and gelatin molecules are adsorbed to the created interface. The aqueous solution is buffered at pH 9; consequently, the bovine protein is negatively charged, making it less suitable for the stabilization of MAA droplets compared to the neutrally charged porcine gelatin [44]. This could lead to bigger droplets decreasing the amount of total interface for the reaction to GelMA, and, therefore decreasing the DoF of both bovine samples. The stabilization mechanism of MAA droplets could explain the missing difference regarding the DoF of GelMA b50 and b225. Additionally, proteins of lower MW show an electrostatic stabilizing effect because of the negatively charged surface, while the stabilizing mechanism of proteins with higher MW is rather steric [41]. As a result, both gelatin types could stabilize the created interface at similar magnitudes, thus showing similar DoF values.

The effect of the MAA-to-gelatin ratio was also studied using four gelatin raw materials, i.e., p80, p175, p300 I, and p300 II. The DoF of each GelMA sample at 40 μL g^−1^ (40 MA) decreased significantly compared to each counterpart at 100 MA (p<0.05). This result is in accordance with similar studies [12,13,20]. Holding the MAA-to-gelatin ratio constant at 40 MA, the DoF did not differ significantly by increasing bloom strength. As the volume of the reactant decreases, the created interface becomes smaller and the stabilization efficiency provided by the proteins is equally effective.

This study shows that the gelatin source as well as bloom strength and even batch-to-batch variations have a significant impact on the process. As the adsorption of the gelatin molecules at the interface to MAA is highly influenced by the MW and MW distribution, the setting of an optimal reactant ratio will depend on the used raw material. Our findings imply process parameters developed using a certain raw material cannot be simply transferred to the operation with a different one. In the case of GelMA, the process parameters to meet a certain DoF have to be adapted according to the gelatin material to be used. Further understanding of the reaction is required taking into account the properties of gelatin at the interface to the reactant. The stirring conditions should also be thoroughly studied, as the droplet size depends on the energy input to the process. As previously stated regarding the use of the protein in the field of tissue engineering, detailed information about the range of operating conditions to meet certain quality attributes is required. This is a requisite by regulatory authorities to reach clinical stages.

### 2.2. Hydrogel Characterization

**Figure 3:**
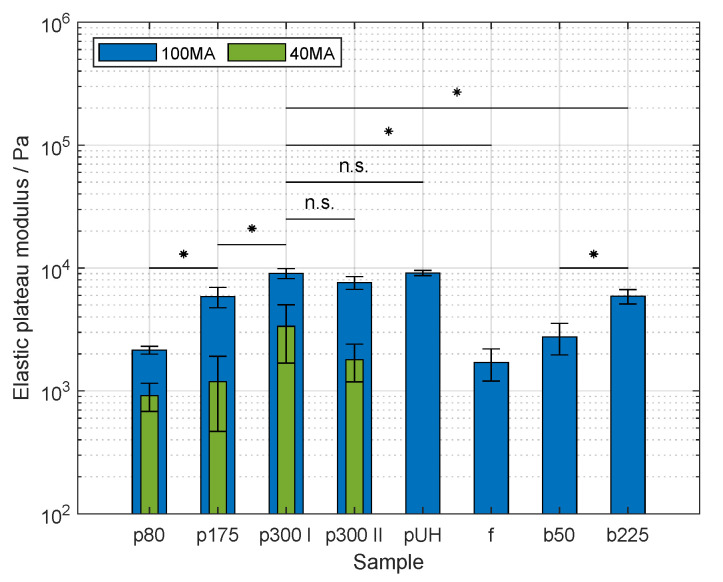
Elastic plateau modulus of gelatin methacryloyl (GelMA) hydrogels of various sources at 10% (*w*/*v*). Sample nomenclature regarding the used raw materials is provided on Table 1. At a methacrylic anhydride (MAA)-to-gelatin ratio of 100 μL g^−1^ (100 MA), asterisks denote a significant difference between synthesized samples (p<0.05). No significant differences are denoted with the abbreviation n.s. (p>0.05). Furthermore, the elasticity of the hydrogels produced with porcine GelMA decreased significantly with decreasing MAA-to-gelatin ratio from 100 to 40 μL g^−1^ (40 MA) (p<0.05). At a MAA-to-gelatin ratio of 40 μL g^−1^ (40 MA), no significant differences regarding the hydrogel elasticity were shown (p>0.05). These differences are not shown for the purpose of clarity. Data are shown as mean and corresponding standard deviation. Each batch of GelMA was used for the formulation of hydrogels. Three samples of each batch were tested.

GelMA solutions can be covalently crosslinked to hydrogels. This possibility is crucial when the intended application takes place at elevated temperatures. As shown in the literature, the elasticity of the produced hydrogels is influenced by the protein concentration and its DoF [11,14,45]. This study aimed to characterize the effect of the source as well as the effect of diverse values of bloom strength of the raw material on the resulting mechanical properties. For this purpose, hydrogels were prepared at 10% (*w*/*v*) as described above, and the storage modulus was determined by oscillatory frequency sweeps on a rheometer. The associated values are presented in Figure 3.

The elastic moduli of the hydrogels prepared with porcine GelMA 100 MA increased significantly with the bloom strength of the respective gelatin raw material (p<0.05). The moduli were 2.15 ± 0.16 kPa, 5.85 ± 1.11 kPa, and 9.04 ± 0.85 kPa, for GelMA samples p80-100 MA, p175-100 MA, and p300 I-100 MA, respectively. The hydrogels produced with p300 II exhibited an elastic modulus of 7.60 ± 0.91 kPa, and the data did not differ significantly from the data produced with p300 I-100 MA, i.e., the same gelatin product used as raw material proceeding from a different batch. Similarly, the elastic moduli of GelMA hydrogels p300 I and p300 II did not differ significantly from that of hydrogels produced with GelMA pUH-100 MA. This effect is in accordance with the work of Aljaber et al. [15]. The study showed an increase in elastic as well as compressive moduli by increasing the bloom of the raw material from 175 g to 300 g. The increasing elasticity corresponds to higher crosslink density in the polymeric network. This resistance to the deformation is influenced by both covalent bonds and physical entanglements [33,46]. GelMA with higher bloom strength showed a higher DoF, and thus a higher amount of methacrylamide and methacrylate residues. Furthermore, as the bloom value of the protein increases, so too does the MW, which leads to an increment in the amount of physical entanglements, as well. Although significant differences in viscosity at the same gelatin concentration were measured meaning a difference in the MW and MW distribution, the missing difference by means of the elasticity of samples p300 I, p300 II, and pUH could arise from the crosslinking conditions in the present study. The irradiation dose was set to 2167 mJ cm^−2^. This condition is much higher than the presented methods in similar studies [11,20,47]. As studied by O’Connell et al. [48], the reaction rate is proportional to the irradiance and photo-initiator concentration in free radical polymerization. As a result, the diffusivity of radicals and the accessibility of crosslinking sites are rapidly lowered by the increasing elasticity of the polymeric matrix, thus limiting the formation of covalent bonds.

Not only the elastic moduli of hydrogels made of porcine GelMA were determined, but also those of fish and bovine GelMA. The samples prepared with f-100 MA, and b50-100 MA showed elastic moduli values of 1.69 ± 0.50 kPa, and 2.75 ± 0.79 kPa, respectively. These data sets were not significantly different from each other. The elastic moduli of b225-100 MA hydrogels had a value of 5.88 ± 0.79 kPa, significantly higher than that of GelMA f-100 MA and b50-100 MA hydrogels (p<0.05). The data measured from the b225-100 MA hydrogel were significantly lower than the data acquired from hydrogels p300 I, p300 II, pUH (p<0.05). The lower elasticity of fish GelMA in comparison to porcine GelMA and bovine GelMA has been shown in the literature. While both Young et al. [18] and Aljaber et al. [15] state the use of porcine gelatin with 300 g bloom strength, only Young et al. mention the bloom value of bovine gelatin, i.e., 225 g. In both cases, hydrogels prepared with fish GelMA show the lowest elasticity. Due to the fact that the DoF of p80-100 MA, f-100 MA, and b50-100 MA were similar, the covalent crosslinks and chain entanglements contribute equally to the elasticity of the hydrogels. Moreover, the effect of increasing elasticity with increasing bloom strength of porcine gelatin is exhibited by the samples prepared with bovine GelMA, as well. As both bovine GelMA samples proved to have a similar DoF, the increasing elasticity of the hydrogel b225-100 MA is a consequence of the larger amount of physical entanglements due to the higher MW of the protein.

The effect of the DoF on the elasticity of hydrogels was characterized using porcine GelMA. The samples prepared with GelMA 40 MA were significantly less elastic than the counterparts produced with GelMA 100 MA (p<0.05). The behavior is attributed to the fact of the lower amount of methacrylamide and methacrylate residues required for photo-crosslinking at an equal GelMA concentration. The effect of DoF on elasticity has been reported in similar studies [14,19,47,48]. Future research should include the characterization of the relationship between the properties of the GelMA backbone, i.e., MW and DoF, and hydrogel properties, i.e., elasticity. Additionally, the protein composition can also be taken into account as the protein sources vary in terms of species. Hence, variability regarding the amount of hydrophilic amino acids along the protein affects the mechanical properties of the hydrogel as well.

### 2.3. Microparticle Generation and Characterization

**Figure 4:**
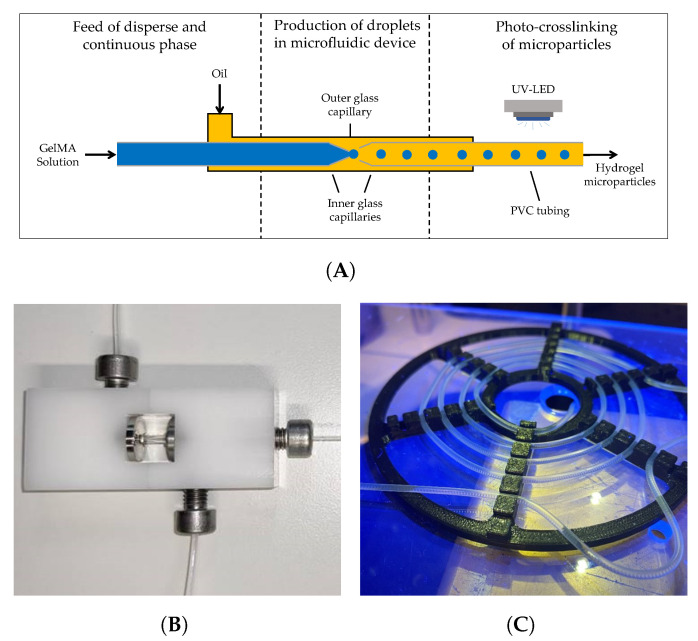
Schematic of the microfluidic setup used in this study. (**A**) Disperse phase and continuous phase, i.e., Gelatin methacryloyl (GelMA) and sunflower seed oil, respectively, were fed into the microfluidic device using a syringe pump. The droplet production took place within an outer glass capillaries, where two inner capillaries were placed. The left and right inner capillaries had diameters of 170 μm and 340 μm, respectively. The dispersed droplets in oil were crosslinked to hydrogel microparticles under ultraviolet (UV) light-emitting diodes (LED). Figure adapted from Leister et al. [53]. The complete experimental setup was used at room temperature. (**B**) Image of the used microfluidic device. (**C**) Image of the light-transmitting tubing under UV irradiance for the crosslinking of hydrogel microparticles.

GelMA has proved to be a versatile material in a wide range of applications, e.g., three-dimensional cell culture in studies of disease and tissue engineering [49]. The formation of physical gels at room temperature imposes a challenge for the manufacturing of GelMA-containing products. Similar to gelatin solutions, GelMA solutions form physical gels due to inter- and intramolecular interactions leading to the formation of helical structures. Regarding the elasticity of physical gels, the effect is less pronounced in GelMA compared to raw gelatin; however, the transition temperature remains that of unmodified gelatin [11]. Because the gelation occurs below physiological temperature, the production of GelMA structures with techniques such as bioprinting [50], electrospinning [51], and microfluidics [52] relies on the addition of further polymers to the formulation in order to adapt the precursor solution to the particular method. Alternatively, the production equipment is heated above the gelation point [25,26]. The aim of this part of the study was the production of GelMA droplets using a microfluidic device at room temperature. For the production of droplets, fish GelMA was dissolved in ultrapure water as the protein solution did not form a physical gel at room temperature. In contrast, porcine GelMA was dissolved in 4 M urea solution to inhibit the gel formation as the process took place at room temperature, i.e., below the gel transition temperature. The processing of GelMA to hydrogel microparticles consists of two consecutive steps. Firstly, GelMA droplets are produced within an oil stream using a microfluidic device. Subsequently, the droplets in oil are covalently crosslinked to hydrogels under UV irradiation as fluid flows within light-transmitting tubing. A schematic draft of the process is provided in Figure 4. The tested samples and corresponding concentrations as well as the feed rates of both continuous and disperse phases are listed in Table 2.

**Figure 5:**
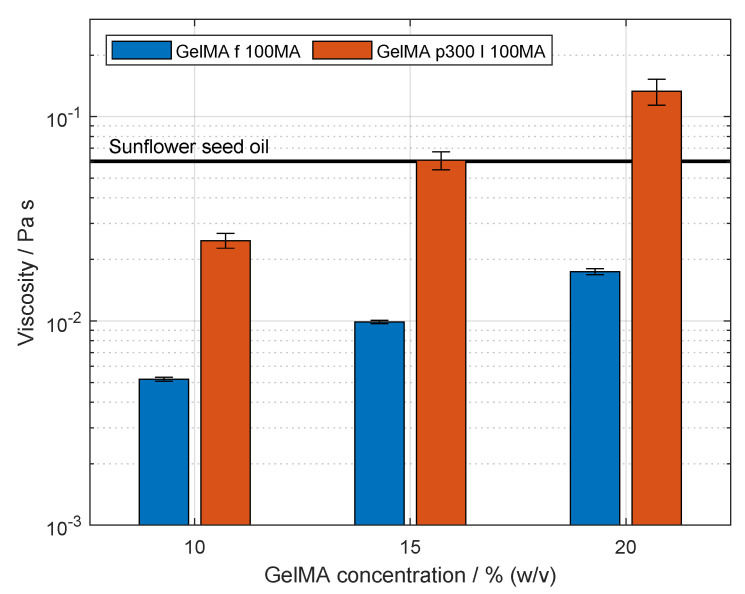
Viscosity of gelatin methacryloyl (GelMA) solutions and sunflower seed oil measured at room temperature. Sample nomenclature is provided in Table 2. The mean value and standard deviation of the viscosity of sunflower seed oil are shown as a black region. The viscosity of oil was measured three times from the same bulk. Fish GelMA was dissolved in ultrapure water, and porcine GelMA was dissolved in 4 M urea solution. The viscosity of both GelMA types was acquired at 10, 15 and 20% (*w*/*v*). The viscosity of the solutions increased significantly with increasing concentration of both types of GelMA (p<0.05). Significant differences were found between the viscosity of the solutions at a constant GelMA concentration (p<0.05). The values are presented as mean and standard deviation. GelMA solutions were measured three times at each concentration. At a constant concentration, GelMA from an independently synthesized batch was used.

As the droplet formation process is influenced by the viscosity of both inner and outer phases, the viscosity of solutions prepared with fish GelMA and porcine GelMA, as well as the viscosity of sunflower seed oil, were measured. The data on the viscosity of GelMA solutions are shown in Figure 5. The viscosity of the oil exhibited a value of 60.5 ± 0.2 mPa s, which is shown as a line across the figure. A significant increase in viscosity was shown with an increasing concentration of both fish and porcine samples, i.e., samples f-100 MA, and p300 I-100 MA, respectively, (p<0.05). The values of viscosity of the fish at the concentrations 10, 15 and 20% (*w*/*v*) were 5.2 ± 0.1 mPa s, 9.9 ± 0.2 mPa s and 17.4 ± 0.6 mPa s, respectively. Solutions containing fish GelMA showed lower values of viscosity compared to those of porcine GelMA at the same protein concentration with values of 24.7 ± 2.0 mPa s, 61.1 ± 6.1 mPa s and 133.0 ± 19.2 mPa s. Furthermore, the viscosity of solutions of both GelMA types at 10% (*w*/*v*) are significantly lower than the viscosity of the solution with the corresponding gelatin product shown in Figure 5.

The effect of increasing porcine GelMA on the viscosity is in accordance with literature [45]. The viscosity of the solution is affected by the amount of bound water which increases with protein concentration. Additionally, the friction between protein chains and the number of physical entanglements of protein chains increases with the concentration [32,33]. The higher values of porcine GelMA solution compared to those of fish GelMA solution have not been reported in the literature, but it is expected since the viscosity of porcine gelatin solutions is higher than that of fish gelatin, as shown above. Additionally, the solution of fish GelMA did not contain urea since fish GelMA does not form a physical gel at room temperature. This fact could also account for the higher viscosity of the porcine GelMA solutions in this study as urea increases the viscosity as well [54]. The decreasing viscosity of the solution after the modification of gelatin to GelMA is in accordance with literature [16,45] and is attributed to the reduction in hydrophilic interaction between the GelMA backbone and the surrounding aqueous phase.

**Figure 6:**
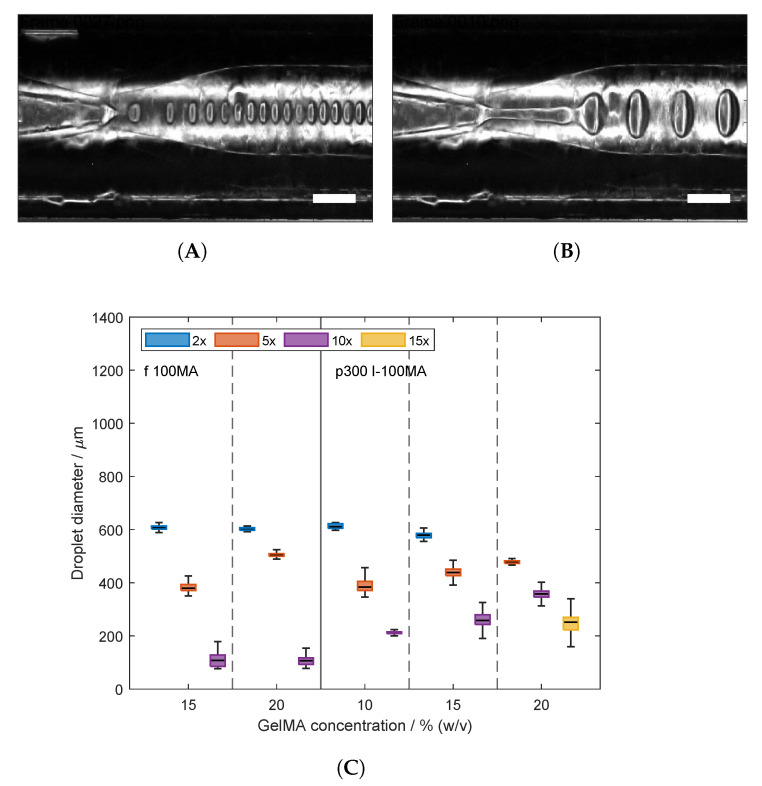
Production of gelatin methacryloyl (GelMA) droplets in a microfluidic device. Fish and porcine GelMA were used in the disperse phase, whereas sunflower seed oil was used in the continuous phase. Sample nomenclature is provided in Table 2. The feed rate of the continuous phase was set to 120 mL min^−1^. (**A**,**B**) Microscopic images of the break-up points of GelMA droplets. Scale bar: 500 μm. (**A**) 15 (*w*/*v*) Fish GelMA with a feed rate of 12 mL min^−1^, i.e., 10× feed ratio. (**B**) 20 (*w*/*v*) Fish GelMA with a feed rate of 24 mL min^−1^, i.e., 5× feed ratio. (**C**) Droplet size of disperse phase composed of fish and porcine GelMA, i.e., samples f-100 MA and p300 I-100 MA, at different concentrations and different feed ratios. The droplet size was measured directly after formation using the “Droplet Morphometry and Velocimetry” (DMV) software [58]. The data were collected from at least 50 droplets of each GelMA sample. Values of the droplet size distribution are shown as a boxplot, where the middle line indicates the median and the edges of the boxes represent the 25 and 75 percentiles. Whiskers indicate maxima and minima within a 1.5-fold interquartile range. Moreover, the droplet size decreased with increasing feed ratio of continuous to disperse phase at each tested composition.

GelMA droplets were produced at the tip of glass capillaries within the microfluidic system. Exemplary images of the droplets at the break-up point are shown in Figure 6A,B. The droplets were formed in a co-flow configuration using the second capillary as a flow restriction to facilitate the droplet formation. Directly after droplet break-up, the particle size was determined using high-speed image acquisition and using automated image analysis to determine the droplet size. The effect of feed ratio as well as GelMA type and concentration were tested. The associated data are shown in Figure 6C. Droplets were generated with fish GelMA at 15 and 20% (*w*/*v*). At both concentrations, increasing the feed ratio led to significantly lower droplet sizes. A similar effect was exhibited in the production of droplets with porcine GelMA solution at room temperature. For GelMA droplets at 10 and 15% (*w*/*v*), the decreasing droplet size was significant. At 20% (*w*/*v*) porcine GelMA, the same trend with respect to the lower concentrations was shown; however, the effect of increasing the feed ratio was not significant. In the literature, GelMA droplets for the production of microparticles have been studied at concentrations up to 10% (*w*/*v*) [23,24,55]. Such low concentration has been used due to the thermal gelation of GelMA solutions at room temperature. Additionally, the heating of the microfluidic devices has been implemented in other studies to maintain the solutions as a liquid [25,26]. In the presented study, droplets of porcine GelMA solution with a protein concentration of 20% (*w*/*v*) could be produced at room temperature. The processing without heating of the devices was feasible due to the presence of urea in the solution. Moreover, the effect of increasing feed rate leading to decreasing droplet size is in accordance with the literature [23,24,26,55]. This is due to the viscous drag of the oil phase in contrast to the decreasing inertial and interfacial force of the disperse phase [56]. Moreover, the study by Wang et al. [25] mentioned the higher droplet size for the solution comprising porcine GelMA compared to the solution containing fish GelMA. Furthermore, the study by Samanipour et al. [24] stated the increasing diameter of particles generated by increasing the GelMA concentration at the same feed rate. These effects were justified as due to an increase in viscosity—the former due to the higher viscosity of the protein of porcine origin and the latter due to the increment of the protein concentration. The droplet formation was in the dripping regime. In our study, this effect of the viscosity on particle size was partially exhibited, but it was not a trend overall. Additionally, the mechanism of droplet formation shifted with increasing viscosity of the protein solutions from dripping to jetting regime as shown in Figure 6A,B, respectively. In our study, the high GelMA concentration lowers the interface tension at a higher magnitude, and therefore the inner phase is more prone to forming a jet stream. Additionally, the viscosity of the oil was not considerably higher than the viscosities of the GelMA solutions. Especially for the sample at 20% (*w*/*v*), where the viscosity of the inner phase exceeds the oil viscosity, the required feed ratios were even higher for the break-up of droplets. These conditions even lead to the widening of the jets, leading to higher droplet sizes [57].

**Figure 7:**
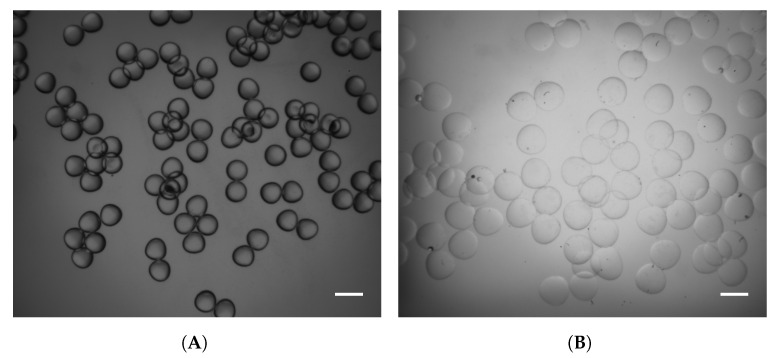

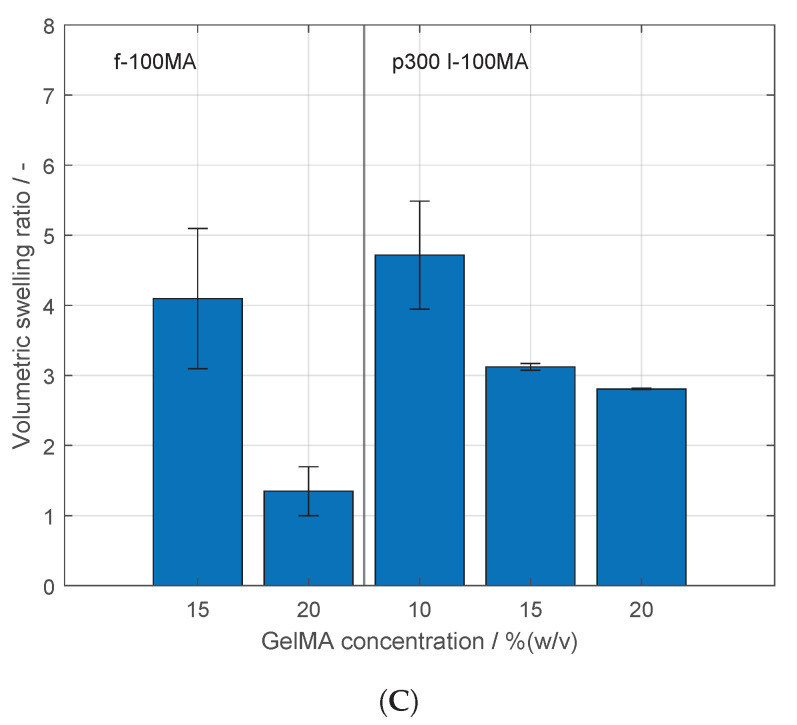
Volumetric swelling ratio of gelatin mathacryloyl (GelMA) microparticles. Fish and porcine GelMA particles were produced at a feed ratio of continuous to disperse phase of 5×. Sample nomenclature is provided in Table 2. (**A**,**B**) Microscopic images of GelMA microparticles. Scale bar: 1000 μm. (**A**) 20 (*w*/*v*) fish GelMA particles collected after photo-crosslinking in the sunflower seed oil phase. (**B**) 20 (*w*/*v*) fish GelMA after swelling to equilibrium in Dulbecco’s phosphate-buffered saline (DPBS). (**C**) Volumetric swelling ratio (VSR) of GelMA particles composed of fish and porcine GelMA, i.e., samples f-100 MA and p300 I-100 MA. The swelling ratio was calculated according to Equation (2). Moreover, the swelling behavior decreased with increasing GelMA concentration of each type. Values are presented as mean and corresponding standard deviation. The particles size for the calculation of the swelling behavior was detected from at least 40 particles of each sample.

Particles generated at a feed ratio of 5× were collected for further analysis regarding the swelling behavior in DPBS. For this purpose, images were taken of the droplets in oil, and after equilibration in DPBS. These images are shown in Figure 7A,B, respectively. The particle diameters in both media were determined using an image processing and analysis workflow developed in Matlab^®^. Moreover, the volumetric swelling ratio was calculated according to Equation (2), and the associated results are shown in Figure 7C. The swelling ratio of fish GelMA particles decreased from 4.10 ± 1.00 to 1.35 ± 0.35 with increasing GelMA concentration. Similarly, the swelling behavior of particles composed of porcine GelMA decreased with increasing protein concentration. The volumetric swelling ratio of the 10% (*w*/*v*), 15% (*w*/*v*), and 20% (*w*/*v*) hydrogel particles were 4.72 ± 0.77, 3.12 ± 0.05, and 2.81 ± 0.01. The effect of GelMA concentration on the swelling capacity of hydrogels has been shown in similar studies [59,60,61]. The swelling process is driven by the osmotic pressure difference between the aqueous phase within the polymeric network and the bulk phase. Counteracting the swelling process is the elasticity of the crosslinked network, which increases with increasing concentration of the protein [1,62]. As mentioned above, the increasing elasticity originates from the higher amount of both covalent bonds and physical entanglements [46]. The presented study shows the production of GelMA droplets and the subsequent crosslinking to hydrogel particles in a single step at room temperature. Fish GelMA and porcine GelMA were used for this purpose, including GelMA concentrations that have not been studied in the literature due to the complexity of the material and its thermal gelation at temperatures below physiological conditions. Further studies regarding droplet production and subsequent crosslinking should include a thorough characterization of the mechanisms of droplet formation including the calculation of dimensionless numbers such as the capillary and Weber number. The droplet formation is influenced by the composition of both phases, which depends on the intended application. Surfactants could be used for the stabilization of GelMA droplets, as the small molecules adsorb rapidly to newly created interfaces, and, hence, avoiding coalescence. For encapsulation of cells as well as biopharmaceuticals, fish GelMA at high concentrations could be used as it can be processed at room temperature without the use of urea as an additive. This is of significant importance as urea induces protein denaturation and cell disruption. Regarding cell delivery, research implies the biocompatibility of used surfactants; therefore, the determination of non-critical concentrations to avoid cytotoxic effects should be included. Furthermore, porcine GelMA can be implemented for the production of microcarriers. As previously reported, cells can attach to the GelMA hydrogels after the purification of GelMA. Hence, hydrogel microparticles can be used for the expansion of adherent cells. This approach increases the area-to-volume ratio of bioreactors compared to the commonly used TC flasks. Similarly, such microcarriers can be implemented for the selective differentiation of stem cells depending on the hydrogel formulation and its stiffness [22,63]. In the present study, the robustness of the production process is increased as the system is not sensitive to temperature fluctuations that could lead to the gelation of the GelMA-containing solutions. Particularly, hydrogel particles at concentrations of 15 and 20% (*w*/*v*) were produced, higher than previously reported in the literature. Hence, stiffer hydrogels could be prepared, which is required for the differentiation and expansion of certain cell phenotypes. Additionally, as GelMA hydrogels can be enzymatically degraded, cells can be easily harvested and separated from the aqueous media. 

## 3. Conclusions

Gelatin methacryloyl is well established for the formulation of hydrogels, finding application in biotechnology, tissue engineering, and biofabrication. The studies on the manufacturing progress have been focused on the use of porcine gelatin as raw material. However, a comparison across the literature of the effects of raw materials on the final product is challenging, as various approaches are employed including differences in the composition of reaction buffer, pH, and buffering capacity. Additionally, the molecular weight of the protein has not been the focus of the reports. In the first part of this study, we produced GelMA at room temperature applying the previously reported method, where urea is used in the reaction buffer to inhibit the thermal gelation of the protein solution. This principle was successfully applied to the operation with a variety of raw materials other than the one used in our previous report. Moreover, insights were gained into the effects of batch-to-batch variability, as two different batches of the same porcine gelatin product were used, and the degree of functionalization of the two products differed. Furthermore, the bloom value, and hence the molecular weight of porcine gelatin, proved to have a significant impact on the degree of functionalization, which decreased with decreasing bloom strength. Additionally, fish gelatin and two bovine gelatin products with varying bloom values were modified to GelMA. The DoF of the products was lower than that of porcine GelMA with high bloom. Our findings underline the significant impact of the raw material on the processing of gelatin to GelMA. As the reactants are not miscible, stirring is required to disperse methacrylic anhydride droplets in the gelatin solution. The protein adsorption at the interface, where the reaction takes place, depends on the molecular weight, molecular weight distribution, as well as protein charge. Therefore, the optimization of process parameters is highly dependent on raw materials, and a developed process cannot simply be transferred to the operation with a different raw material. Further research should take into account the properties of gelatin at the interface to the reactant. Furthermore, the produced GelMA materials of varying species and bloom strength were used for the formulation of hydrogels, and the elasticity of the polymeric network was characterized. By variation in the degree of functionalization, GelMA hydrogels showed increasing elastic moduli. In addition, the molecular weight of the raw materials affected the elasticity. Decreasing the bloom strength of GelMA hydrogels led to less elastic behavior.

As a second part of the presented study, two GelMA types were used for the microfluidic generation of droplets and the subsequent crosslinking to hydrogel particles. Both processes were performed on a single pipeline at room temperature. Therefore, fish GelMA was dissolved in water and porcine GelMA was dissolved in urea solution to maintain a solution at room temperature. The droplets could be produced at higher GelMA concentrations than that found in the literature. Moreover, the droplet size decreased with an increasing feed ratio of the continuous to disperse phase at each tested concentration. The swelling behavior of crosslinked particles was characterized. Hydrogel particles exhibited a higher swelling degree with decreasing GelMA concentration. Future studies should include the formulation of GelMA using surfactants as well as the adaptation of the modular microfluidic device to stabilize the droplets by other means. Additionally, further understanding of the mechanisms of GelMA droplet generation is required, specifically how process parameters affect dripping and jetting regimes.

## 4. Materials and Methods

### 4.1. Synthesis and Characterization of Gelatin-Methacryloyl

#### 4.1.1. Precursor Solution for the Synthesis of Gelatin-Methacryloyl

Gelatin products were purchased from Sigma-Aldrich (St. Louis, MI, USA), and the relevant product information is listed in Table 1. The buffer for the dissolution and synthesis of GelMA was prepared following the method by Grijalva Garces et al. [14]. Buffer components for the synthesis of GelMA were acquired from Merck (Darmstadt, Germany). The buffer composition was 0.25 M carbonate bicarbonate (CB) and 4 M urea. After the dissolution of the salts, the pH of the solution was adjusted to pH 9 using 1 M sodium hydroxide (NaOH) or 1 M hydrochloridic acid (HCl). Gelatin was dissolved in the synthesis buffer to a concentration of 10% (*w*/*v*) at room temperature under stirring.

#### 4.1.2. Rheological Characterization of Gelatin Solutions

The viscosity of the solutions at 10% (*w*/*v*) gelatin in synthesis buffer was measured using a rotational rheometer (Physica MCR301, Anton Paar, Graz, Austria). For the characterization of the gelatin solutions, the configuration of the rheometer included a cone-plate geometry (diameter 60 mm, cone angle 0.5°), and a solvent trap in order to avoid sample drying during the measurements. The viscosity was determined within the shear rate range of 0.5 to 500 s^−1^.

The viscosity values of the gelatin solutions provided below were determined as triplicates. Each value was measured from an independently prepared solution. Values are shown as mean and standard deviation.

#### 4.1.3. Synthesis and Purification

The synthesis was performed at room temperature under stirring. The reaction was started by adding methacrylic anhydride (MAA, 94%, Sigma-Aldrich) to the gelatin solutions. The MAA-to-gelatin ratio was 100 μL g^−1^ for all gelatin samples. Additionally, porcine gelatin was modified to GelMA with a ratio of 40 μL g^−1^. Throughout this study, sample nomenclature includes 100 MA, and 40 MA depending on the used MAA-to-gelatin ratio. The reaction was carried out for 60 min. The process was terminated by two-fold dilution with ultrapure water and a subsequent pH adjustment to pH 7.4. The diluted reaction mixture was then dialyzed with a3.5 kDa molecular weight cut-off tubing (Thermo Fisher Scientific, Waltham, MA, USA) in an ultrapure water reservoir. This purification took place for 4 days at 40 °C. GelMA solutions were frozen at −80 °C overnight and lyophilized. Solid GelMA samples were stored at room temperature until further use.

#### 4.1.4. Determination of Degree of Functionalization

The degree of functionalization (DoF) of GelMA samples was determined based on the method by Habeeb [38]. Therefore, glycine (Sigma-Aldrich), gelatin materials, and GelMA samples were dissolved in ultrapure water. Glycine standards for the determination of a standard curve were prepared at 3, 5, 8, 10 and 20 μg mL^−1^. Gelatin references and GelMA samples were dissolved in ultrapure water at 0.1, 0.3, 0.5 and 0.8 mg mL^−1^. A 0.1 M CB buffer at pH 8.5 was used as a reaction buffer containing 0.01% (*w*/*v*) trinitrobenzenesulfonic (TNBS) acid (Sigma-Aldrich). A volume of 250 mL of the TNBS reagent solution was mixed with an equal volume of the gelation as well as GelMA samples. Incubation followed for 2 h at 40 °C. The reaction was terminated by addition of 250 μL of a 10% (*w*/*v*) sodium dodecyl sulfate (Sigma-Aldrich) solution and 125 μL of a 1 M HCl solution. A microplate reader (infiniteM200, Tecan Group, Männedorf, Switzerland) for the measurement of the sample absorbance at 335 nm. The concentration of free amines in the samples was determined in comparison to a glycine calibration curve and normalized to the respective gelatin concentration. The DoF was calculated according to Equation (1). The difference between the number of free amino groups present in gelatin ($c_{NH_{2},gelatin}$), i.e., before the functionalization, and the amount in the produced GelMA ($c_{NH_{2},GelMA}$), i.e., after the reaction, was divided by the number of free amines in the raw gelatin.

(1) $$\mathrm{Degree}\;\mathrm{of}\;\mathrm{Functionalization}\%=\frac{c_{NH_{2},gelatin}-c_{NH_{2},GelMA}}{c_{NH_{2},gelatin}}$$

The production and characterization of GelMA samples consisted of three experimental runs using each gelatin source. The measurement of absorbance in order to determine the DoF was performed for each independently synthesized batch. The values are shown as mean and standard deviation.

### 4.2. Hydrogel Characterization

#### 4.2.1. Precursor Solution for the Production of Hydrogels

GelMA samples synthesized from different sources and MAA-to-gelatin ratios were used for hydrogel preparation. For this purpose, a solution containing 0.1% (*w*/*v*) the photo-initiator lithium phenyl-2,4,6-trimethylbenzoylphosphinate (LAP, Sigma-Aldrich) was prepared in Dulbecco’s phosphate-buffered saline (DPBS, without calcium and magnesium, 1×, pH 7.4, Thermo Fisher Scientific). Subsequently, the lyophilized material was dissolved to 10% (*w*/*v*) in LAP containing DPBS at 40 °C. GelMA solutions were transferred to cylindrical polytetrafluoroethylene (PTFE) molds (diameter 10 mm, height 3 mm) by pipetting a volume of 235 μL. The samples were then crosslinked to hydrogels by exposure to an ultraviolet (UV) light-emitting diode (LED, 365 nm, OSRAM, Munich, Germany) with an irradiation intensity of 12 mW cm^−2^ for 3 min. GelMA hydrogels were equilibrated in DPBS until further analysis.

#### 4.2.2. Mechanical Characterization

The viscoelastic properties of hydrogels were characterized using a rotational rheometer Physica MCR301. A plate-plate geometry (diameter 10 mm) and a solvent trap were part of the configuration of the rheometer. The cylindrical hydrogels were placed on the bottom plate and the top plate was positioned to a gap height of 2.5 mm. Storage and loss moduli were measured within the linear viscoelastic (LVE) regime covering the frequency range of 0.5 to 50 rad s^−1^. A constant stress amplitude was set to 0.5 Pa. The data of the mechanical characterization were acquired from three experimental runs with three samples each. For each run, GelMA hydrogels from independently produced batches were prepared. The data are presented below as the mean and standard deviation of the elastic plateau modulus.

### 4.3. Microparticle Fabrication and Characterization

#### 4.3.1. Precursor Solution for the Production of Microparticles

The manufacturing of GelMA hydrogel microparticles at room temperature was investigated. For these experiments, porcine GelMA, i.e., p300 I, and fish GelMA at a reactant ratio of 100 μL MAA per gram gelatin were used. A 4 M urea solution was prepared for the dissolution of porcine GelMA. Subsequently, the photoinitiator LAP was dissolved to 1% (*w*/*v*). Lyophilized porcine GelMA was added to the mixture and dissolved under stirring. The precursor solution containing fish GelMA was prepared by dissolving the lyophilized material in ultrapure water containing 1% (*w*/*v*) LAP. Samples from both sources were prepared at 10, 15 and 20% (*w*/*v*). The precursor solutions were protected from light prior to their use in the disperse phase in the microfluidic device as described below. The tested samples and the used concentrations are summarized in Table 2. Sunflower seed oil (Sigma-Aldrich) was employed as continuous phase.

#### 4.3.2. Rheological Characterization of Disperse and Continuous Phase

The shear-rate dependent viscosity of sunflower seed oil and the GelMA solutions was characterized as described in Section 4.1.2. For these measurements, the solvent trap served additionally as protection from light to avoid photo-crosslinking during the measurement.

The viscosity of the oil phase was measured as triplicates from the same bulk. The value is presented as the mean and associated standard deviation. Regarding the rheological characterization of the GelMA solutions, the viscosity was determined at each concentration as triplicates. Each value was acquired from the prepared solution from each independently synthesized batch.

#### 4.3.3. Microfluidic Production of Droplets and Crosslinking to Microparticles

The setup for the production of GelMA droplets and the subsequent crosslinking process is shown schematically in Figure 4A. The disperse and continuous phases, i.e., GelMA solutions and sunflower seed oil, respectively, were filled in high-precision glass syringes (SETonic, Ilmenau, Germany). A Nemesys syringe pump was used to control the feed rates using the software QmixElements v20140605 (both CETONI, Korbussen, Germany). The rate of the oil phase was set to a constant value of 120 mL min^−1^ for all experiments. The tested GelMA concentrations and the corresponding feed rates, as well as the feed ratio, defined as the ratio of the feed rate of the continuous phase to that of the disperse phase, are listed in Table 2.

For the formation of GelMA droplets, a microfluidic device with glass capillaries was employed, as shown in Figure 4B. A detailed description of the equipment is provided by Leister et al. [53]. The setup consisted of one outer and two inner glass capillaries (World Precision Instruments, Friedberg, Germany). The inner capillaries (outer diameter 1 mm, inner diameter 0.58 mm) were modified by pulling with a micro-pipette puller (P-1000, Sutter Instruments, Novato, CA, USA). The tip diameter of the capillary for the disperse phase was 170 μm, while the tip diameter of the second capillary used as the outlet of dispersed droplets in oil was 340 μm. The inner capillaries as well as the outer capillary (length 15 mm, inner diameter 1.56 mm) were treated with 2-[methoxy(polyethyleneoxy)6-9-propyl]tris(dime thylamino)silane (Gelest Inc., Morrisville, PA, USA) in order to render the surface hydrophobic. The capillaries were attached in the polyoxymethylene (POM) module as published by Bandulasena et al. [64]. The distance between inner capillaries was set to 170 μm. The outlet of the microfluidic device was connected to a polyvinyl chloride (PVC) tubing (outer diameter 1.8 mm, inner diameter 1 mm, Deutsch & Neumann, Hennigsdorf, Germany). The tubing was arranged as a loop under four UV LEDs (OSRAM), where photo-crosslinking took place with a total irradiation intensity of 25.6 mW cm^−2^. An image of the tubing placed under UV light is provided in Figure 4C. The produced hydrogel microparticles in oil were collected and stored at room temperature until further analysis.

#### 4.3.4. Determination of Droplet Size

Image sequences of the formation of droplets at the break-up point were acquired using a monochrome camera (DMK 33U, The Imaging Source Europe, Bremen, Germany) equipped with a 1× lens (TMN 1.0/50, The Imaging Source Europe) using the software IC capture V2.5 (The Imaging Source Europe). The acquisition rate was set to 10 frames per second. The resulting droplet sizes were determined using “Droplet Morphometry and Velocimetry” (DMV) software [58] by analyzing at least 150 frames. The coordinates of the center of each droplet, as well as the respective droplet diameter in each frame, were exported. Detected objects with a center below or above the longitudinal axis of the capillaries at the direct proximity of the break-up point were considered outliers and removed from the distributions. The data distribution of at least 50 droplets per sample is shown below as box plots including median, upper, and lower quartile, as well as maxima and minima within a 1.5-fold interquartile range.

#### 4.3.5. Determination of Hydrogel Swelling Behavior

For the determination of the swelling behavior of the hydrogel microparticles, the samples from both GelMA sources produced with a feed ratio of 5× were collected. DPBS was added to the particle/oil mixture and centrifuged at 500 rcf. The excessive oil and DPBS were removed keeping the particles in the bottom of the centrifuge tube. The microparticles were suspended in fresh DPBS and centrifuged. This wash series was performed four times. Microparticles were then equilibrated overnight in DPBS. Hydrogel microparticles and the corresponding oil phase and DPBS were placed on a microscopy slide for image acquisition. The imaging setup consisted of a monochrome camera (Genie Nano M2420 Mono, Teledyne Dalsa, Waterloo, ON, Canada) equipped with a 10× objective (Nikon, Tokyo, Japan). For the quantification of the particle size, an image processing and analysis workflow was developed using Matlab^®^ R2023a (TheMathWorks Inc., Natick, MA, USA) with the library Image Processing Toolbox 11.7. The variation in pixel intensities compared to the nine-by-nine surrounding pixels was analyzed using the local entropy filter. The output images contain high-intensity values in the regions of high-intensity variation, i.e., the interface between microparticles and bulk media. Particle diameters were detected on said output processed images. The swelling behavior of the hydrogel microparticles was characterized by the ratio of media diameter after swelling in DPBS ($d_{p,DPBS}$) to the median diameter in oil ($d_{p,Oil}$), as shown in Equation (2). The data sets presented below correspond to the swelling ratio of at least 40 hydrogel particles of each sample.

(2) $$\mathrm{Volumetric}\;\mathrm{Swelling}\;\mathrm{Ratio}\%=\frac{d_{p,DPBS}^{3}}{d_{p,Oil}^{3}}$$

### 4.4. Data Handling and Statistical analysis

Image processing, data evaluation, data visualization, and statistical analysis of the data sets were performed with Matlab^®^ R2023a (TheMathWorks Inc., Natick, MA, USA). One-way analysis of variance (ANOVA) was performed in order to determine significant differences. A *p*-value below 0.05 was considered as statistically significant.

## Figures and Tables

**Table 1:** Overview of gelatin types for the synthesis of gelatin methacryloyl (GelMA). The products were purchased from Sigma-Aldrich; the corresponding product information is provided including source, Bloom strength is according to the manufacturer, as is the sample nomenclature used throughout this manuscript.

Product Number	Batch Number	Source	Bloom Strength	Nomenclature
G6144	SLCH4483	porcine	80–120 g	p80
G2625	SLCC4273	porcine	175 g	p175
G1890	SLCC7838	porcine	300 g	p300 I
G1890	SLBX2973	porcine	300 g	p300 II
39465	BCBW7164	porcine	ultrahigh	pUH
G7765	038K0681	fish	–	f
G6650	SLCM1231	bovine	50–120 g	b50
G9382	SLCF9893	bovine	225 g	b225

**Table 2:** Composition of disperse phase as employed for the production of hydrogel microparticles as well as the feed rate and feed ratio of continuous phase to disperse phase. The feed rate of the continuous phase consisting of sunflower seed oil was set to 120 mL min^−1^.

GelMA Sample	Concentration % (*w*/*v*)	Feed Rates mL min^−1^	Feed Ratios *x*
f-100 MA	15	12, 24, 60	10, 5, 2
f-100 MA	20	12, 24, 60	10, 5, 2
p300 I-100 MA	10	12, 24, 60	10, 5, 2
p300 I-100 MA	15	12, 24, 60	10, 5, 2
p300 I-100 MA	20	8, 24, 60	15, 10, 5

## Data Availability

The raw data supporting the conclusions of this article as well as the written codes for Matlab^®^ will be made available on request. Inquiries can be directed to the corresponding author.

## Abbreviations

The following abbreviations are used in this manuscript:

CB	Carbonate-Bicarbonate
DoF	Degree of functionalization
DPBS	Dulbecco’s phosphate-buffered saline
ECM	Extracellular matrix
GelMA	Gelatin-Methacryloyl
IEP	Isoelectric point
LED	Light-emitting diode
MAA	Methacrylic anhydride
MW	Molecular weight
RT	Room Temperature
TC	Tissue culture
TE	Tissue engineering
TNBS	2,4,6-Trinitrobenzenesulfonic acid solution
UV	Ultraviolet
VSR	Volumetric swelling ratio
